# AI-driven personalised management platform enhances glycaemic control and remission rates in type 2 diabetes: a quasi-experimental prospective cohort study

**DOI:** 10.3389/fdgth.2026.1778918

**Published:** 2026-05-07

**Authors:** Reziwanguli Amuti, Areziguli Kadier, Mengting Jiang, Abudureheman Maihemuti, Bingquan Yang

**Affiliations:** 1 Endocrinology Department, Zhongda Hospital, Southeast University, Nanjing, Jiangsu, China; 2 Department of Respiratory Medicine, Shenzhen Hengsheng Hospital, Shenzhen, Guangdong, China; 3 Department of Gynecology, People's Hospital of Bayingol Mongolian Autonomous Prefecture, Korla, Xinjiang, China; 4 School of Public Health, Southeast University, Nanjing, Jiangsu, China; 5 Musculoskeletal Research Laboratory, Department of Orthopaedics and Traumatology, Prince of Wales Hospital, The Chinese University of Hong Kong, Hong Kong, Hong Kong SAR, China; 6 Department of Endocrinology, Zhongda Hospital Lishui Branch, Southeast University, Nanjing, Jiangsu, China

**Keywords:** AI-driven management platform, diabetes management, DSME, personalized management care, type 2 diabetes

## Abstract

**Background and aim:**

Although artificial intelligence (AI) technologies show promise in diabetes management, real-world evidence supporting AI-driven personalized care remains limited. We developed an AI-powered platform that generates dynamic self-management plans (DSMPs) and enables continuous, whole-process patient monitoring. This study aimed to evaluate its effectiveness in patients with type 2 diabetes mellitus (T2DM).

**Design:**

This was a 12-month, multicenter, prospective, open-label, quasi-experimental cohort study. A total of 1,452 eligible patients with T2DM were enrolled between April 2022 and April 2023 and assigned by an AI-driven platform, based on enrollment sequence, to either the intervention group (*n* = 726) or the control group (*n* = 726). All participants received integrated diabetes management via the AI-driven platform. The intervention group was provided with DSMPs, whereas the control group received routine care.

**Findings:**

Among the 1,343 patients who completed the study (655 in the intervention group and 688 in the control group), the intervention group demonstrated significantly better glycemic control at 3 months. Reductions in glycated hemoglobin (HbA1c) (1.92% vs. 1.31%) and fasting plasma glucose (FPG) (3.23 vs. 2.31 mmol/L) were significantly greater in the intervention group, and significantly more patients achieved treatment targets for both HbA1c (57% vs. 26%) and FPG (68% vs. 39%). Furthermore, the intervention led to significant improvements (all *p* < 0.05) in Self-Monitoring of Blood Glucose (SMBG) frequency, hypoglycemia incidence, Body Mass Index (BMI), Urine Albumin-to-Creatinine Ratio (UACR), and most blood lipids compared to the control group and baseline, though Total Cholesterol (TC) remained unchanged (*p* = 0.53). Notably, the co-primary outcome was achieved by 18.2% (119/655) of patients in the intervention group, which was significantly higher than the 5.5% (38/688) observed in the control group (*p* < 0.001).

**Conclusions:**

AI-driven personalized management was feasible and effective for the management of T2DM and was associated with an increased rate of diabetes remission. However, further evidence is required to justify its integration into routine clinical practice, and improvements in cholesterol management remain necessary.

## Introduction

Diabetes mellitus (DM) is one of the most common and increasingly prevalent chronic diseases, affecting an estimated 424.9 million individuals in China Among these individuals, only 32.7% receive treatment, and only about half achieve adequate glycemic control (1–3). DM and its complications pose a significant threat to individual health and impose a substantial financial burden on families and society (4–7). Diabetes self-management education and support (DSME/S) has been recognized as a foundation for assisting patients in daily decision-making and self-care activities, with demonstrated benefits for health outcomes (8, 9). However, barriers such as limited access to quality education, unequal distribution of medical resources, and a shortage of diabetes educators—especially in rural areas—have contributed to a low diabetes awareness rate of only 36.7% in China (10). Moreover, patients often struggle to acquire sufficient self-management knowledge during limited outpatient consultation time.

The use of personalized care plans has been shown to improve health outcomes and care experiences among individuals with T2DM (11). Diabetes self-management is a continuous process. While DSME/S enhances inpatient education and post-discharge adherence, it often fails to provide continuous support for outpatients. With advances in internet technology, a variety of digital applications have been developed to support diabetes management (12–15). Furthermore, a growing body of evidence suggests that digital health interventions can augment traditional care for patients with T2DM (16).

Recent advances in artificial intelligence (AI) have opened new avenues for the management of chronic diseases, particularly diabetes. AI- and machine learning-based approaches have demonstrated substantial potential in predicting glycemic variability (17, 18), delivering personalized interventions, and supporting sustainable behavior change (15, 19). However, despite these promising applications, large-scale real-world studies evaluating the long-term clinical benefits of deeply integrating AI into regionalized, end-to-end diabetes management programs remain scarce, especially with respect to diabetes remission.

To address this gap, we developed an AI-driven Diabetes Management Platform, a comprehensive chronic disease management system designed to enable seamless data exchange both within hospital settings and across public health systems. The platform incorporates machine learning algorithms to continuously analyze patient-generated data, including blood glucose levels, dietary intake, physical activity, and medication adherence. Based on predefined rules and predictive AI models, the system automatically generates personalized dynamic self-management plan (DSMP) recommendations. In addition, it provides data-driven clinical decision support to assist clinicians in optimizing and finalizing individualized management plans, while offering early warnings for acute risks such as hypoglycemia and hyperglycemia.

In this study, we implemented a regionalized, comprehensive, and personalized diabetes management model—namely, the AI-driven personalized DSMP—and evaluated its feasibility and effectiveness in patients with type 2 diabetes mellitus (T2DM).

## Methods

### Study design

This was a 12-month, multicenter, prospective, open-label, quasi-experimental cohort study conducted across Nanjing Lishui People's Hospital and seven collaborating community health service centers within the Southeast University integrated diabetes management and research program. The study protocol was approved by the Ethics Committee of Southeast University (2021ZDYLL370-P01).

### Study participants

Participants were recruited from inpatients diagnosed with T2DM in the Departments of Endocrinology and Metabolism of the participating hospitals between April 2021 and April 2022.

#### Inclusion and exclusion criteria

Inclusion criteria: (1) Diagnosis of T2DM, aged 18–85 years; (2) Medical records established at Lishui District People's Hospital or one of the seven participating community hospitals; (3) Provision of written informed consent; (4) Ability to use smartphones to record blood glucose, blood pressure, dietary intake, and physical activity data; to submit inquiries; to access self-management materials; and to communicate with healthcare providers.

Exclusion criteria: (1) Severe or active cardiovascular or cerebrovascular diseases; (2) Malignant tumors or severe hepatic or renal insufficiency (aminotransferase levels >3× the upper limit of normal or chronic renal insufficiency ≥ stage IV); (3) History of psychiatric disorders or cognitive impairment that could compromise data collection; (4) Pregnancy or planned pregnancy during the study period; (5) Disabilities or paralysis interfering with study follow-up; (6) Other active chronic diseases (e.g., asthma) or active medical conditions (e.g., peptic ulcer); (7) Deemed unsuitable for participation by the investigators.

#### Research tools

##### AI-driven diabetes management system (AI-DMS)

**Figure 1:**
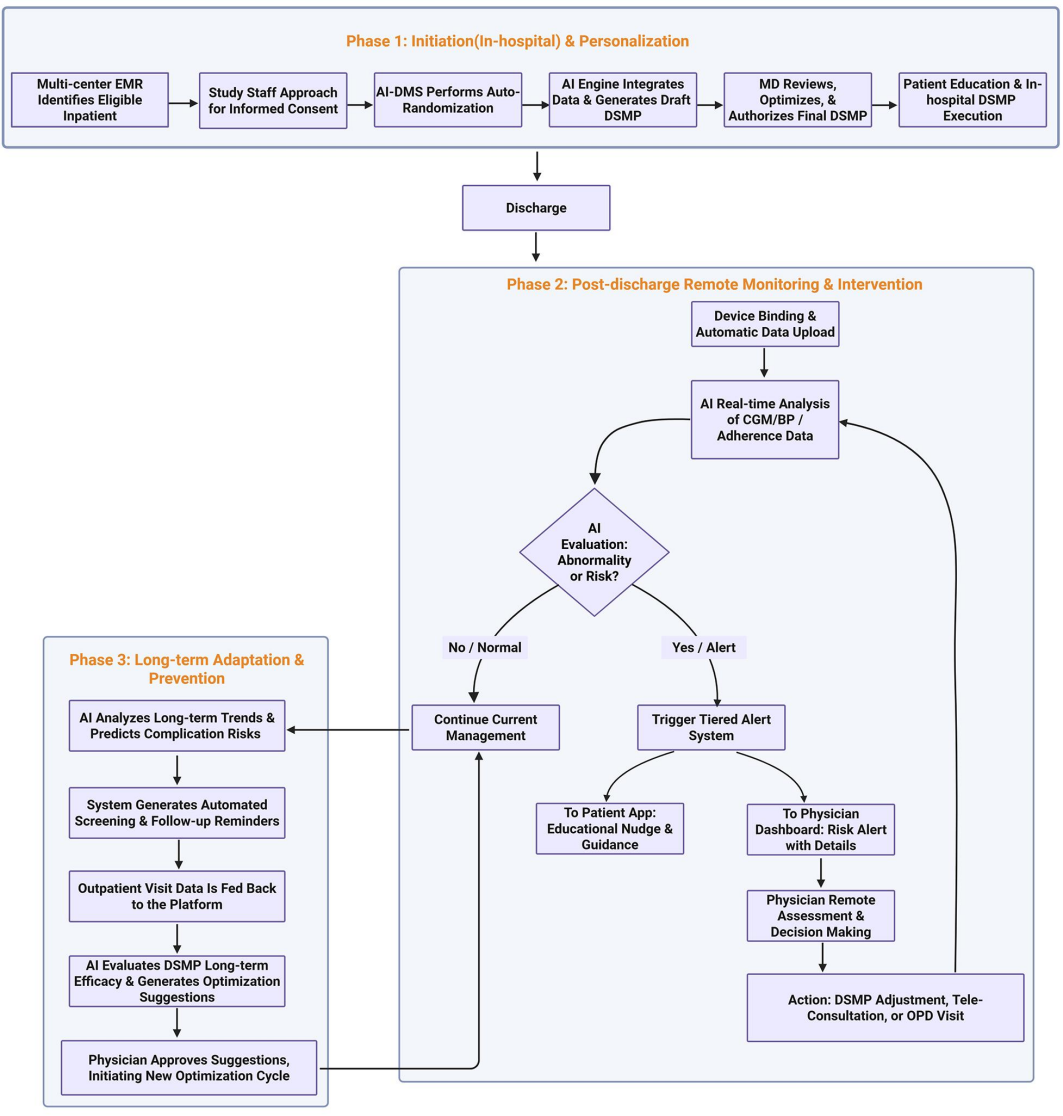
Graphical illustration of the functions of the AI-driven diabetes management system.

Study data were collected and managed using the AI-DMS, a secure, web-based platform developed by Zhongda Hospital and its Lishui Branch. The system integrates multiple AI technologies to enable comprehensive diabetes management (Figure 1).

#### AI analysis engine architecture

**Core Technologies:** The platform employed a hybrid AI approach that combined rule-based reasoning systems with machine learning models, including predictive models and clustering algorithms for patient stratification. **Data Processing:** The analysis engine continuously processed and analyzed structured data from multiple sources, including patient-entered data (blood glucose, blood pressure, dietary logs, exercise records, and medication adherence), electronic medical records, and laboratory results. **Real-time Analytics:** Streaming data processing was implemented to enable immediate analysis of incoming patient data, facilitating proactive identification of potential risks.

#### Personalized management plan generation

**Initial DSMP Creation:** Based on a comprehensive assessment of patients with T2DM conducted during hospitalization or the initial outpatient visit, the AI analysis engine automatically generated a preliminary personalized DSMP.

**Multi-domain Optimization:** The generated DSMPs provided specific and actionable recommendations across multiple domains.

**Glycemic Management:** Personalized glycemic targets, recommended SMBG frequency (e.g., times per week), and optimal testing time points (preprandial, postprandial, or bedtime) were determined based on the patient's current glycemic status and medication regimen. For example, intensified monitoring was recommended following insulin initiation to mitigate hypoglycemia risk, whereas monitoring frequency was reduced during periods of stable glycemic control. **Dietary Management:** Individualized dietary recommendations were generated by calculating total daily caloric intake and macronutrient distribution based on patient height, body mass index, current glycemic control, and metabolic parameters, including lipid and uric acid levels. Exercise Regimen: Exercise prescriptions specified recommended frequency, intensity, and appropriate types of physical activity, tailored to individual weight loss goals and comorbid conditions. Adjustments were made for patients with conditions such as coronary heart disease or asthma. Goal Alignment: All recommendations were tailored to achieve individualized management targets based on patient-specific characteristics, including age, sex, disease duration, presence of complications, and predefined treatment objectives.

#### Clinical decision support with physician oversight

**Human-in-the-Loop Optimization:** Attending physicians reviewed AI-generated DSMP drafts through the web-based platform. The system prompted physicians to approve or manually override the plan based on unstructured clinical information, such as recent changes in dietary habits or the presence of newly diagnosed comorbidities (e.g., coronary heart disease). Owing to the efficiency of this interface, physicians could accept the AI-generated plan with a single click when no adjustments were required. Consequently, the rates of acceptance or rejection were not separately tracked, as the system was designed for seamless integration into routine clinical workflows. **Final Authorization:** All DSMPs required physician approval before electronic delivery to patients, ensuring clinical validity and patient safety.

#### Dynamic adaptation mechanism

**Iterative Optimization:** The AI engine continuously monitored patient-reported outcomes and clinical metrics. At each subsequent visit or upon changes in a patient's clinical condition, the system automatically generated a revised DSMP based on updated information, including glycemic control, body weight changes, and laboratory results. This dynamic update mechanism ensured that the management plan evolved in accordance with patient progress over time.

#### Predictive alert system

**Pattern Recognition:** AI models identified abnormal patterns, including sustained hyperglycemia trends, increased risk of frequent nocturnal hypoglycemia, and patterns indicative of medication non-adherence. **Proactive Intervention:** Automated alerts were sent to healthcare providers when potential risks were identified, while educational push notifications were concurrently delivered to patients. For example, real-time analysis of uploaded glucose data enabled the system to immediately notify both clinicians and patients upon detection of critically abnormal values.

##### Dynamic self-management plan (DSMP)

Developed in accordance with the Expert Consensus on Self-management Plan for Type 2 Diabetes Mellitus in China (2017 Edition), the DSMP integrated comprehensive diabetes management domains. In this study, the DSMP served as the structured output of the AI-DMS, translating personalized AI-generated recommendations into a clear and actionable schedule for patients. The DSMP encompassed key management components, including glycemic control, exercise regimen, dietary management, and goal alignment.

**Figure 2:**
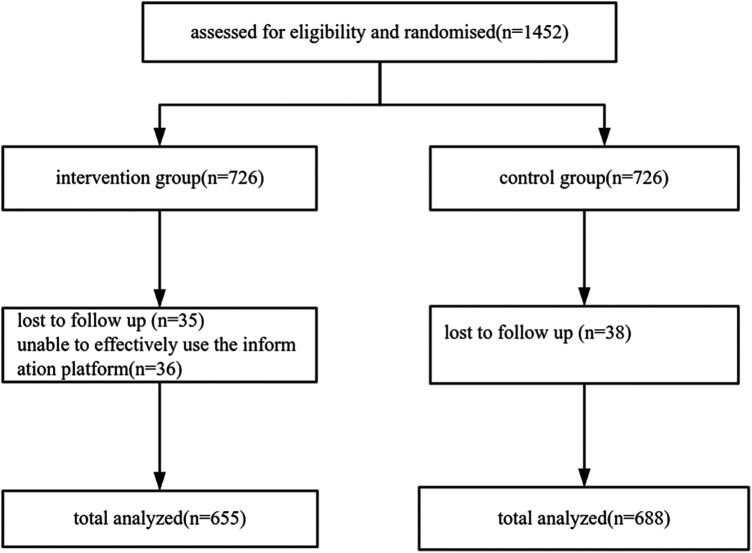
Trial registration flowchart.

**Procedures and randomization.** Eligible inpatients were identified and received standardized counseling from local staff or study nurses in the endocrinology departments of the participating institutions. Written informed consent was obtained from all participants. Participants were allocated to either the intervention group or the control group in a 1:1 ratio based on whether the trailing digit of their electronic filing number was odd or even. This allocation method constituted a quasi-random approach rather than true random sequence generation. Owing to the nature of the intervention and the allocation procedure, blinding of participants and clinical staff was not feasible, and allocation concealment was not implemented (Figure 2).

#### Intervention and control

Research nurses or general practitioners guided all participants in adding the “Chronic Disease Doctor” WeChat applet, completing real-name registration using personal identification and mobile phone numbers, and entering basic patient information. Comprehensive instructions on the applet's functions and usage were provided.

Control group: Participants in the control group received routine care throughout the 12-month study period. Routine care included standard medical consultations, face-to-face T2DM education, lifestyle counseling, and access to the “Chronic Disease Doctor” applet. For this group, the applet functioned primarily as a tool for recording self-monitoring data (via manual entry or device linkage for blood glucose and physical activity), accessing diabetes-related educational materials, and reviewing examination results. Reminder settings were available only for manually entered data. Medication adjustments and routine face-to-face diabetes self-management education (DSME) were provided during clinical visits based on examination findings and/or self-monitoring data recorded in the applet.

Intervention group: Participants in the intervention group received all components of routine care in addition to an AI-generated personalized DSMP. Following a comprehensive assessment of the patient's condition during hospitalization or an outpatient visit, the AI-DMS generated an initial DSMP. These plans were reviewed and optimized by physicians before being delivered electronically through the applet, with supplementary paper copies provided for patient reference. Notably, the applet for the intervention group was configured to actively implement the DSMP by providing visualized reminders for SMBG according to the AI-recommended monitoring frequency and timing.

Outcome measures: Primary outcomes included glycated hemoglobin (HbA1c), fasting plasma glucose (FPG), and body mass index (BMI). Secondary outcomes included triglycerides (TG), total cholesterol (TC), low-density lipoprotein cholesterol (LDL), high-density lipoprotein cholesterol (HDL), urine albumin-to-creatinine ratio (UACR), incidence of hypoglycemia, and frequency of self-monitoring of blood glucose (f-SMBG).

Data were collected at baseline and at 12 months post-intervention. HbA1c and FPG were additionally assessed at quarterly follow-up visits. Analyses compared mean values, rates of achievement of predefined treatment targets, and co-primary outcomes. Subgroup analyses were conducted according to sex and age groups (young: ≤35 years; middle-aged: 36–60 years; elderly: ≥61 years).

Treatment targets: The predefined treatment targets were as follows: FPG, 4.4–7.0 mmol/L; HbA1c ≤ 7.0%; TG ≤1.7 mmol/L; TC ≤4.5 mmol/L; LDL ≤2.6 mmol/L; HDL ≥1.0 mmol/L for males and ≥1.3 mmol/L for females; and f-SMBG ≥80% (at least 83 measurements per year).

To evaluate therapeutic effects across different metabolic parameters, we calculated the attainment rate (also referred to as the control rate) for each predefined treatment target. The attainment rate represents the proportion of patients who achieved the specified goal for a given metabolic indicator, thereby providing a detailed profile of treatment efficacy for individual outcomes. The attainment rate for each target was calculated separately as follows:

$$\mathrm{Attainment}\;\mathrm{Rat}e_{x}=\frac{\mathrm{Number}\;\mathrm{of}\;\mathrm{patients}\;\mathrm{achieving}\;\mathrm{target}\;x}{\mathrm{Total}\;\mathrm{number}\;\mathrm{of}\;\mathrm{patients}\;\mathrm{in}\;\mathrm{the}\;\mathrm{group}}\times 100\text{\%}$$

These individual attainment rates were subsequently presented in Figure 5 to facilitate a clear comparison of which specific metabolic goals were more readily achieved between the different treatment groups.

Co-primary outcomes (indicative of diabetes remission potential) were defined as the simultaneous achievement of the following criteria: 1) HbA1c < 6.5%; 2) BMI ≤ 24 kg/m^2^, or weight loss ≥10% over 12 months for patients with baseline BMI > 24 kg/m; 3) Follow-up adherence ≥80%.

### Statistical analysis

Data were managed using Microsoft Excel and analyzed with SPSS version 25.0 (IBM Corp., Armonk, NY, USA). The normality of continuous variables was assessed using the Shapiro–Wilk test in combination with visual inspection of Q–Q plots. Normally distributed continuous variables are presented as means with standard deviations (SDs), whereas categorical variables are presented as frequencies and percentages.

Baseline demographic and clinical characteristics were compared between the intervention and control groups using independent *t*-tests for continuous variables and *χ*^2^ tests for categorical variables.

Changes in HbA1c and FPG from baseline across four follow-up time points were analyzed using two-way repeated-measures analysis of variance (ANOVA), with group (intervention vs. control) and time as the two factors. The main effects of group and time, as well as the group-by-time interaction, were examined. When a significant interaction effect was detected, *post hoc* pairwise comparisons with Bonferroni correction were performed to adjust for multiple comparisons.

For age subgroup analyses, to determine whether the intervention effect differed across age categories, a two-way ANOVA was conducted with group and age category as fixed factors and the change in HbA1c or FPG from baseline to the specified follow-up time point as the dependent variable. The group-by-age interaction was tested. Baseline characteristics across age groups were compared using one-way ANOVA for continuous variables and *χ*² tests for categorical variables.

All statistical tests were two-tailed, and statistical significance was defined as a *p* value < 0.05.

## Results

### Baseline characteristics of participants

A total of 1,452 patients were enrolled in this study, of whom 110 were lost to follow-up (71 in the intervention group and 39 in the control group). Ultimately, 1,343 patients completed valid observations, including 655 patients in the AI-personalized management group (intervention group) and 688 patients in the conventional information platform group (control group).

Baseline demographic and clinical characteristics were generally well balanced between the two groups. No significant differences were observed in disease duration, BMI, HbA1c, FPG, lipid profiles, or UACR (all *p* > 0.05). The only exception was a slightly higher proportion of males in the control group (*p* = 0.03) (Table 1).

**Table 1:** Baseline characteristics of patients between two groups.

Variable	Intervention group (*n* = 655)	Control group (*n* = 688)	*p*
Sex			
Male	304 (46.4)	376 (54.7)	0.03
Female	351 (53.6)	312 (45.3)	
Age (years)	61.1 ± 11.8	59.9 ± 13.0	0.086
Young (45≤	52	84	
Middle (46∼65)	361	349	
Old (≥65)	242	255	
Disease course	9.2 ± 7.3	8.5 ± 7.3	0.1
BMI (kg/m^2^)	25.13 ± 3.07	25.38 ± 3.33	0.163
HbA1c [% (mmol/mol)]	10.0 ± 1.95	9.91 ± 2.07	0.425
FPG (mmol/L)	11.18 ± 4.72	11.08 ± 5.10	0.699
TG (mmol/L)	3.17 ± 3.81	3.16 ± 2.22	0.960
TC (mmol/L)	4.79 ± 1.02	4.87 ± 1.92	0.154
LDL (mmol/L)	3.21 ± 1.09	3.26 ± 1.08	0.384
HDL (mmol/L)	1.27 ± 0.28	1.27 ± 0.29	0.91
UACR (mg/g)	104.55 ± 72.41	101.65 ± 49.84	0.455

Disease course: years.

### Evaluation of treatment effects

Following the 12-month intervention period, patients in the intervention group demonstrated significantly greater improvements in glycemic control, self-management behaviors, and metabolic parameters compared with those in the control group.

### Glycemic control trajectories

**Figure 3:**
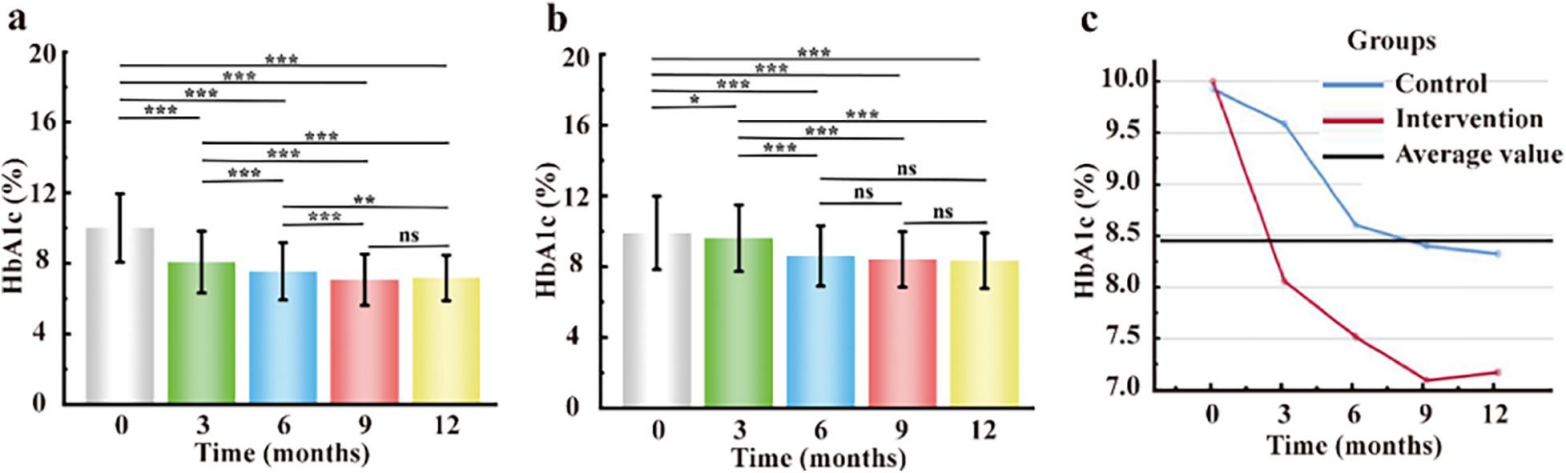
Treatment effects on HbA1c: **(a)** Within-group comparison in the intervention group. **(b)** Within-group comparison in the control group. **(c)** Time × group interaction and between-group comparisons. *p < 0.05; **p < 0.01; ***p < 0.001; ns, not significant (*p* > 0.05).

The intervention group exhibited a rapid and sustained reduction in glycated hemoglobin (HbA1c), with the most pronounced decrease observed at the first follow-up (Δ = −1.92%). Near-target HbA1c levels (approximately 7%) were achieved by the end of the study period. In contrast, the control group showed a delayed response, with the maximum reduction occurring at the second follow-up (Δ = −1.31%), and HbA1c levels subsequently stabilizing at suboptimal values (8.4–8.6%) (Figures 3a,b). Furthermore, a two-way repeated-measures analysis of variance (ANOVA), with group (intervention vs. control) and time (baseline, 3, 6, and 12 months) as factors, revealed a significant group-by-time interaction effect on HbA1c levels (*F* = 43.185, *p* < 0.001) (Figure 3c).

**Figure 4:**
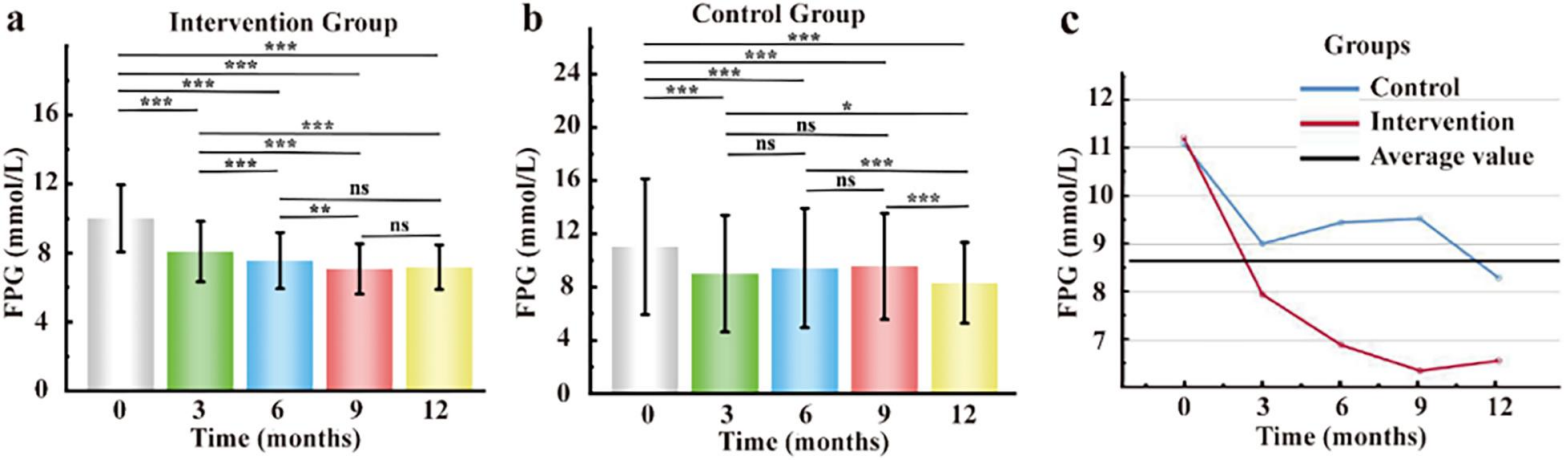
Treatment effects on fasting plasma glucose (FPG). **(a)** Within-group comparison in the intervention group. **(b)** Within-group comparison in the control group. **(c)** Time × group interaction and between-group comparisons. *p < 0.05; **p < 0.01; ***p < 0.001; ns, not significant (*p* > 0.05).

Similarly, for fasting plasma glucose (FPG), both groups exhibited initial improvements. However, only the intervention group achieved and maintained target FPG levels (4.4–7.0 mmol/L), whereas the control group demonstrated rebound hyperglycemia, with FPG levels ranging from 8.3 to 9.4 mmol/L during follow-up (Figures 4a,b). Consistently, a two-way repeated-measures analysis of variance (ANOVA), with group (intervention vs. control) and time (baseline, 3, 6, and 12 months) as factors, revealed a significant group-by-time interaction effect on FPG (*F* = 42.639, *p* < 0.001) (Figure 4c).

**Figure 5:**
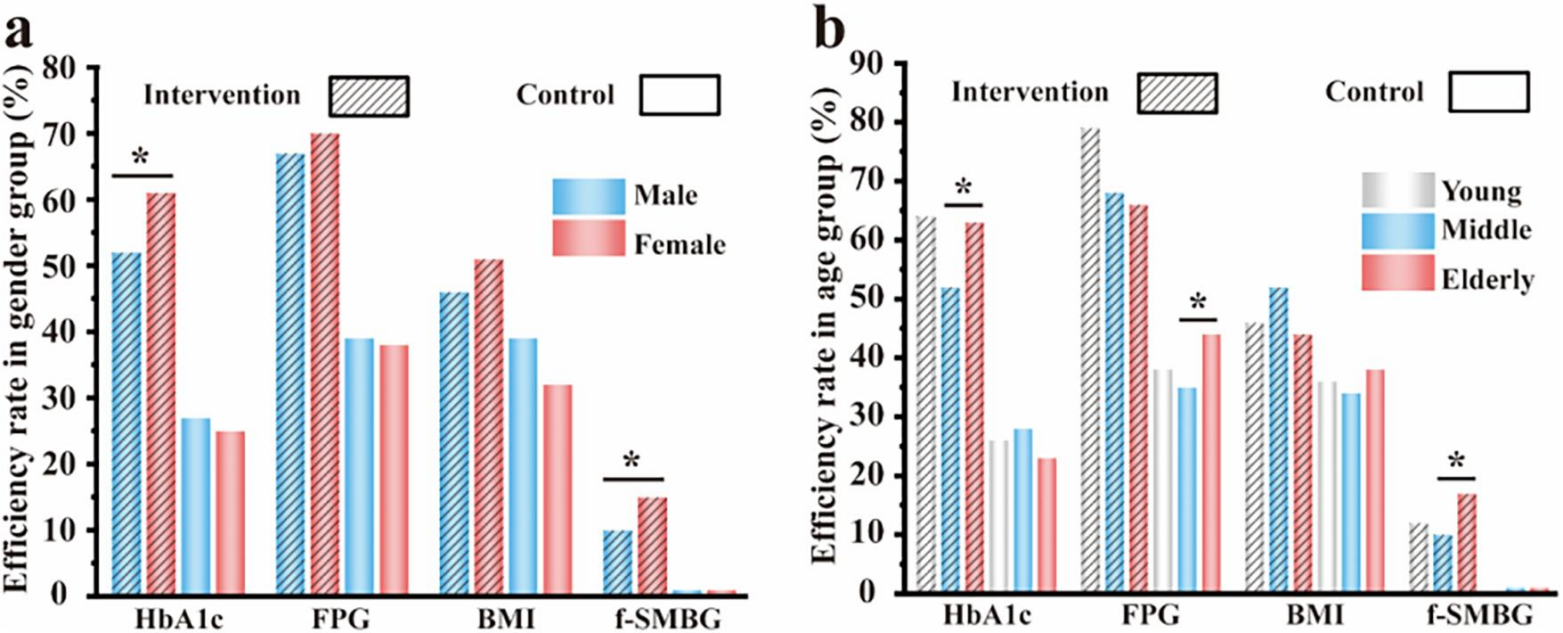
Treatment efficacy rate: **(a)** evaluation of treatment efficacy rate in gender subgroup after intervention; **(b)** evaluation of treatment efficacy rate in age subgroup after intervention; percentage = number of patients achieving the treatment target divided by the total number of patients in the corresponding subgroup × 100%. * indicates *p* < 0.05 for comparisons between subgroups.

### Metabolic and renal parameters

The AI-personalized management group demonstrated comprehensive metabolic improvements, with significant reductions in BMI, UACR, TG, and LDL, along with a significant increase in HDL (all *p* < 0.05). TC remained unchanged (*p* = 0.53). In contrast, the control group showed limited metabolic benefits, with significant worsening of both UACR and TC (*p* < 0.05), and no significant improvements in BMI, LDL, or HDL (Supplementary Table S1).

### Treatment efficacy rate

The AI-personalized management group achieved significantly higher treatment target attainment rates across multiple metabolic parameters compared with the control group (all *p* < 0.05) (Supplementary Table S2).

### Gender-specific analysis

Intervention Group: At baseline, no significant differences were observed between men and women in the attainment rates of HbA1c, FPG, BMI, TG, TC, or LDL (all *p* > 0.05), with the exception of HDL, which was significantly higher in men than in women (84% vs. 43%, *p* < 0.0001). This marked difference in HDL attainment persisted throughout the study period. At 12 months, women in the intervention group achieved significantly higher HbA1c target attainment than men (61% vs. 52%, *p* = 0.026). Conversely, HDL attainment remained significantly higher in men than in women (85% vs. 56%, *p* < 0.0001). No other significant sex-based differences were observed for FPG, BMI, TG, TC, LDL, or f-SMBG at 12 months (all *p* > 0.05).

Control Group: At baseline, no significant gender differences were detected for most parameters, with the exception of TC, for which women exhibited a higher attainment rate than men (50% vs. 42%, *p* = 0.043), and HDL, which was significantly higher in men than in women (89% vs. 43%, *p* < 0.0001). At 12 months, men in the control group demonstrated significantly higher attainment rates for TC (19% vs. 13%, *p* = 0.042) and LDL (34% vs. 26%, *p* = 0.023). HDL attainment remained markedly higher in men than in women (66% vs. 21%, *p* < 0.0001). A trend toward higher BMI attainment was observed in men, although this did not reach statistical significance (39% vs. 32%, *p* = 0.065). No significant gender differences were found for HbA1c, FPG, TG, or f-SMBG (both 1%; Fisher's exact test, *p* > 0.05) (Supplementary Table S2, Figure 5a).

### Age-stratified analysis

Within the intervention group, overall comparisons revealed significant differences in 12-month attainment rates for HbA1c (*p* = 0.004), HDL (*p* < 0.001), and f-SMBG (*p* = 0.045). After Bonferroni correction, the following pairwise comparisons remained statistically significant: for HbA1c, middle-aged vs. elderly patients (52% vs. 63%, *p* = 0.009); for HDL, young vs. middle-aged patients (79% vs. 44%, *p* < 0.001) and elderly vs. middle-aged patients (65% vs. 44%, *p* < 0.001); and for f-SMBG, elderly vs. middle-aged patients (17% vs. 10%, *p* = 0.013). No other pairwise comparisons reached statistical significance (all *p* ≥ 0.0167). Overall differences in attainment rates for FPG, BMI, TG, TC, and LDL were not significant (all *p* > 0.05).

In the control group, overall comparisons revealed significant differences in attainment rates at month 12 for TC (*p* = 0.023) and HDL (*p* = 0.023). After Bonferroni correction, only the comparison between young and middle-aged patients for TC (26% vs. 15%, *p* = 0.011) and between middle-aged and elderly patients for HDL (50% vs. 39%, *p* = 0.006) remained statistically significant. The pairwise difference observed for FPG between elderly and middle-aged patients (44% vs. 35%, *p* = 0.021) did not meet the corrected significance threshold. No other significant age-related differences were detected for HbA1c, BMI, TG, LDL, or f-SMBG (all *p* > 0.05). (Supplementary Table S2, Figure 5a).

### Diabetes remission indicators

A key finding was that 119 patients (18%) in the AI-personalized management group achieved the composite endpoint indicative of diabetes remission potential—defined as HbA1c < 6.5%, significant weight control, and high follow-up adherence—compared with only 38 patients (6%) in the control group (*p* < 0.001) (Table 2). Subgroup analyses further showed that middle-aged patients (19%) and female patients (19%) in the intervention group achieved the highest remission rates.

**Table 2:** Co-primary outcomes: 1) HbA1c < 6.5% (mmol/mol); 2) BMI ≤24 kg/m², or weight loss ≥10% over 12 months for patients with baseline BMI >24 kg/m²; 3) follow-up adherence ≥80%.

Variable	Intervention (*n* = 655)	Control (*n* = 688)
Co-primary outcomes	18% (119)	6% (38)
Male	17% (50)	6% (22)
Female	19% (69)	5% (16)
Young	17% (9)	2% (2)
Middle	19% (67)	6% (21)
Elderly	18% (43)	6% (15)

## Discussion

This study demonstrates the feasibility and effectiveness of an AI-driven personalized dynamic self-management plan (DSMP) for the management of type 2 diabetes. We observed that mean HbA1c and fasting plasma glucose (FPG) levels at all four follow-up time points were significantly lower in the intervention group than in the control group (*p* < 0.05). Notably, after 12 months, 57% (*n* = 373) of patients in the intervention group achieved HbA1c levels below 7.0%, which was significantly higher than the 27% (*n* = 179) observed in the control group. In addition, the intervention group exhibited significantly lower rates of missed follow-up visits, a reduced incidence of hypoglycemia, and improved adherence to f-SMBG (20). Collectively, these findings indicate that the AI-personalized DSMP approach is superior to conventional management strategies for glycemic control (21).

An important observation of this study was that although the basic AI-driven platform management alone (control group) produced early improvements in glycemic outcomes, its long-term effects were limited, which is consistent with previous reports (22). In contrast, the AI-driven personalized DSMP demonstrated sustained effectiveness throughout the entire study period (23, 24). Repeated-measures ANOVA further revealed a significant interaction effect between intervention group and time for both FPG and HbA1c, indicating that the AI-personalized approach resulted in progressively greater improvements in glycemic control over time (25).

The core advancement of the present approach lies in its AI-driven personalization mechanism. Unlike static educational content or passive data-recording platforms, the AI system continuously analyzes real-time patient-generated data streams to deliver dynamic, adaptive feedback and individualized plan adjustments (26, 27). This capability likely underlies the sustained patient engagement and improved metabolic outcomes observed in the intervention group, effectively mitigating patient fatigue commonly associated with fixed management regimens. Furthermore, by efficiently processing multidimensional data, the AI algorithms distill actionable insights for both clinicians and patients, addressing the challenge of information overload during time-limited clinical consultations. The system's AI-supported risk prediction and early warning functions may also partly explain the observed reduction in hypoglycemic events (27).

Stratified analyses by age and sex revealed important patterns in treatment outcomes. Age-stratified analyses indicated that elderly patients derived the greatest glycemic benefits, while both younger and older adults achieved higher HbA1c target attainment rates than middle-aged participants. A statistically significant difference was observed between middle-aged and elderly patients (52% vs. 63%, *p* = 0.009), which may reflect competing demands—such as work and family responsibilities—that limit engagement with structured management programs among middle-aged individuals. Consistently, this group also demonstrated lower adherence to structured f-SMBG compared with elderly patients (10% vs. 17%, *p* = 0.013). Gender-stratified analyses further showed that the AI-driven personalized DSMP particularly enhanced outcomes among female patients. Women in the intervention group achieved significantly higher HbA1c target attainment rates than men at 12 months (61% vs. 52%, *p* = 0.026), despite the absence of baseline differences. Although the basic platform intervention alone appeared to confer greater benefits in male patients (28), the incorporation of AI-driven personalization substantially improved glycemic control in women. This finding suggests that the tailored DSMP may effectively address gender-specific barriers in diabetes self-management.

The importance of weight management in T2DM is well established (29), with evidence indicating that weight loss exceeding 10% can favorably influence disease progression and reduce long-term complications (30). In the present study, the AI-driven approach effectively supported weight control through visualized self-management content and goal-oriented feedback, leading to higher rates of target weight achievement and prevention of UACR progression. In contrast, the basic platform intervention alone did not result in significant changes in BMI, which is consistent with previous findings reported by Hesseldal et al. (31).

Improvements in lipid profiles further highlight the comprehensive benefits of the AI-driven personalized management strategy. The intervention group achieved a mean reduction of 0.47 mmol/L in LDL, with 50% of patients reaching target levels, accompanied by significant improvements in HDL and TG. In contrast, no significant change in TC was observed, which may be partly attributable to dietary compensation occurring alongside improved glycemic control, a possibility that warrants further investigation (32). Sex- and age-stratified analyses further revealed differential responses to the AI-personalized intervention with respect to lipid control. In the control group, male patients demonstrated more favorable lipid outcomes under the basic platform intervention, consistent with previous reports. Notably, a pronounced and persistent disparity in HDL attainment was observed, with men consistently outperforming women across all time points (85% vs. 56%, *p* < 0.0001). This finding suggests the influence of underlying biological factors that may be relatively resistant to current intervention strategies. Similarly, an age-related disparity in HDL attainment was identified, with middle-aged individuals showing lower achievement rates compared with both younger and elderly participants. This pattern mirrors the observed sex-specific differences and points to complex, interrelated physiological and behavioral factors that merit further investigation.

Most notably, the intervention group demonstrated a significantly higher rate of achieving the composite outcome indicative of diabetes remission potential (17% vs. 5%), with particularly promising results observed among female and younger patients. Together with the substantial improvements in glycemic control and body weight, these findings strongly suggest that the AI-driven comprehensive intervention model holds unique potential for promoting type 2 diabetes mellitus remission. This observation aligns with contemporary evidence emphasizing weight management as a central therapeutic strategy in diabetes care (33–36).

Compared with existing literature, this study makes several distinct contributions. Whereas prior research has reported limited effectiveness of basic digital health platforms for diabetes management, our findings demonstrate that the integration of an AI-driven personalization engine can generate substantial additional clinical value. Moreover, while many previous AI-focused studies in diabetes have concentrated on isolated components—such as prediction models or decision-support tools—our work advances the field by presenting a fully integrated platform embedded within a regional healthcare system. The physician–AI collaborative framework, validation through a 12-month quasi-experimental cohort study, and the inclusion of remission-related composite endpoints together represent meaningful advancements beyond prior reports.

### Strengths and limitations

This study has several notable strengths. First, the large-sample real-world design and 12-month intervention period enhance both the external validity and the clinical relevance of our findings. Second, the streamlined AI-assisted workflow improved patient adherence while simultaneously increasing clinical efficiency. Third, the fully integrated regional diabetes management model enabled real-time self-management education and facilitated cross-system connectivity, thereby improving information sharing and optimizing healthcare resource utilization.

Nevertheless, several limitations should be acknowledged. First, health-economic outcomes were not evaluated, which is particularly relevant in the context of recent drug pricing reforms in China. Second, data on certain diabetes-related complications, comorbidities, and patient-reported satisfaction metrics were unavailable. Third, potential confounding factors associated with long-term diabetes management—such as broader healthcare disruptions related to the COVID-19 pandemic—were not fully accounted for. Fourth, the one-year follow-up period limited our ability to assess longer-term clinical outcomes and sustainability of benefits.

With specific regard to the AI system itself, detailed algorithmic specifications, including model architectures and feature engineering strategies, were not exhaustively described. Future studies should aim to provide greater technical transparency where feasible. In addition, performance metrics of the AI components—such as prediction accuracy and recommendation effectiveness—were not independently evaluated within the present clinical effectiveness framework. Dedicated algorithm-validation studies are therefore warranted. Finally, the mechanisms by which AI-personalized content and dynamic plan-adjustment logic influence patient adherence remain incompletely understood and should be further explored using qualitative and mixed-methods approaches.

## Conclusions

The AI-driven personalized DSMP demonstrated significant advantages over conventional approaches in the management of T2DM. This comprehensive intervention was associated with marked improvements in glycemic control, lipid profiles, body weight, and renal parameters, alongside reductions in hypoglycemic events and enhancements in patient adherence. Notably, the substantial rate of achievement of the composite outcome indicative of diabetes remission suggests genuine potential for disease modification rather than merely risk-factor control. Collectively, these findings support both the effectiveness and feasibility of AI-driven personalized diabetes management and justify its broader clinical application. Future implementation efforts should place particular emphasis on optimizing cholesterol management algorithms and systematically addressing the limitations identified in this study.

Future research should focus on further refinement of AI algorithms, particularly those targeting cholesterol management and demographic subgroups with heterogeneous responses. Integration of additional data sources—such as continuous glucose monitoring (CGM) systems and wearable activity trackers—may enhance personalization and predictive accuracy. Expanding AI applications to include complication prediction and medication optimization represents another promising direction. Moreover, longer-term follow-up studies (3–5 years) are needed to confirm the sustainability of diabetes remission and to evaluate impacts on hard clinical endpoints. Comprehensive cost-effectiveness analyses will be essential to inform health policy and large-scale adoption. Finally, improving transparency and explainability of AI decision-making processes will be critical for strengthening clinician trust and facilitating safe, responsible integration into routine diabetes care.

## Data Availability

The original contributions presented in the study are included in the article/Supplementary Material, further inquiries can be directed to the corresponding authors.
